# Simultaneous Therapy with Intravitreal Dexamethasone Implant and Bevacizumab for the Treatment of Macular Edema

**Published:** 2016

**Authors:** Felipe L. de ANDRADE, Flavio S. LOPES, Gabriel C. de ANDRADE, Tiago S. PRATA, André MAIA

**Affiliations:** 1 Retina Clinic, Rua Eloi Cândido Lopes, 291 Centro, Osasco - SP, 06010-130, Brazil; 2 Federal University of São Paulo, Rua Sena Madureira, 1500 - Vila Mariana, São Paulo - SP, 04021-001, Brazil

**Keywords:** Bevacizumab, Macular Edema, Retinal Vein Occlusion, Immunosuppression, Dexamethasone

## Abstract

To investigate the safety profile and benefits of a short-term simultaneous treatment regimen combining two drugs—an intravitreal implant of dexamethasone with an intravitreal injection of bevacizumab—in patients with macular edema. This was a retrospective, non-randomized, open-label case series study. Patients were treated between April 2014 and July 2015 and were diagnosed with recurrent macular edema secondary to diabetic retinopathy and retinal vein occlusion. They underwent simultaneous treatment with an intravitreal injection of bevacizumab (1.25 mg) and an intravitreal implant of dexamethasone (0.7 mg). Patients were evaluated at baseline and at each subsequent visit with a complete ophthalmological examination and spectral-domain optical coherence tomography (OCT) scans. They were examined 24 hours after the treatment, and then followed up after 30 days and 60 days. Twenty patients (representing 20 eyes) were included in the study. At the time of injection (i.e., baseline), the best-corrected visual acuity (BCVA) was 0.758 ± 0.42 logarithm of the minimum angle of resolution (logMAR). It improved significantly to 0.51 ± 0.33 logMAR at 1 month and to 0.5 ± 0.34 logMAR at 2 months (P ≤ 0.03). The median baseline central macular thickness (CMT) was 542 µm (interquartile range, 466 – 751 µm). The median CMT decreased significantly to 321 µm (interquartile range, 288–381 µm) at 1 month and 310 µm (interquartile range, 286 – 354 µm) at 2 months (P ≤ 0.0002). The mean intraocular pressure (IOP) increased from 14.9 ± 2.29 mmHg (at baseline) to 16.5 ± 2.99 mmHg (P = 0.04) after 2 months. Two (10%) eyes showed cataract progression. There were no other ocular or systemic complications for the duration of this study. Simultaneous therapy combining a dexamethasone implant plus bevacizumab for macular edema may be an attractive treatment regimen with an acceptable safety profile.

## INTRODUCION

Macular edema (ME) occurs in many ocular pathologies (e.g., diabetic retinopathy and vein occlusion) and causes different degrees of visual impairment. Pathogenesis could be related to inflammatory cytokines (e.g., interleukin-6 and prostaglandin), dysregulation of endothelial tight junction proteins, and increased vascular endothelial growth factor (VEGF) expression (1-5). The expression of inflammatory factors breaks down the blood–retinal barrier, and thereby causes capillary leak, fluid accumulation, and increased macular thickness (3-5). Managing ME may involve laser therapy, intravitreal injections of anti-VEGF agents, or corticosteroid preparations. Several pharmacological treatment regimens have been introduced to treat ME such as intravitreal bevacizumab (Avastin; Genentech, Inc., South San Francisco, CA), ranibizumab (Lucentis; Genentech, Inc.), aflibercept (Eylea; Regeneron, Inc., Tarrytown, NY, USA), and a biodegradable intravitreal implant that slowly releases the corticosteroid dexamethasone (Ozurdex; Allergan, Inc, Irvine, CA) (3, 6). These pharmacological therapies have made it possible to improve vision rather than just stabilize it, and may significantly improve a patient’s quality of life (4). Anti-VEGF drugs decrease the concentration of free VEGF, but do not interfere with other proinflammatory molecules that mediate vascular permeability. Corticosteroids block the production of VEGF and other inflammatory mediators (5, 7). The combination of a corticosteroid with anti-VEGF drugs may substantially suppress vascular permeability and consequently decrease macular thickness, improve visual acuity, and increase the interval between injections, compared to the outcomes obtained with either of these medications alone—especially in eyes that present with severe edema (2, 3). The aim of this pilot study was to investigate the safety profile and benefits (i.e., improved visual acuity and decreased macular thickness) of short-term simultaneous treatment with an intravitreal injection of bevacizumab (1.25 mg) and intravitreal dexamethasone implant (0.7 mg) in patients with ME.

## MATERIALS AND METHODS

This retrospective case series was a non-randomized, open-label, single-center investigation (Retina Clinic, São Paulo, Brazil). It was approved by the Institutional Review Board of Hospital Oftalmológico de Sorocaba/SP (São Paulo, Brazil) and adhered to the tenets of the Declaration of Helsinki. Study participants were patients older than 18 years who were treated between April 2014 and July 2015, and diagnosed as having recurrent ME secondary to diabetic retinopathy and retinal vein occlusion. They underwent simultaneous treatment with an intravitreal injection of bevacizumab (Avastin [1.25 mg]; Genentech, South San Francisco, CA) and an intravitreal dexamethasone implant (Ozurdex [0.7 mg]; Allergan, Inc, Irvine, CA). Patients with macular edema had a central macular thickness greater than 300 µm, based on spectral-domain optical coherence tomography (OCT). For this study, a variation greater than 6 mmHg in intraocular pressure (IOP) was considered abnormal. Key exclusion criteria were previous treatment with intravitreal anti-VEGF within 6 weeks and previous treatment with laser or intravitreal corticosteroids within 3 months of study entry.

 Patients were evaluated at baseline and at each subsequent visit with best-corrected Snellen visual acuity (BCVA) evaluation, slit-lamp examination, gonioscopy, indirect examination, intraocular pressure (IOP) measurement (Goldmann applanation tonometry), and spectral-domain OCT (Zeiss Cirrus, Dublin, CA).

The procedure was performed in an operating room under sterile conditions. Two minutes before the injections, the patient received an initial drop of proparacaine (0.5%) onto the study eye, followed by 5% povidone-iodine solution. The eyelid margins, the eyelids and the periocular skin were then washed with povidone-iodine. The eye was draped in a sterile manner. A sterile lid speculum was inserted by the surgeon. A subconjunctival bleb of anesthesia was created by injecting 0.4 mL of lidocaine (1%) in the superior temporal quadrant. A caliper was used to mark the injection site 3.5 mm from the limbus in pseudophakic eyes and 4.0 mm in phakic eyes. A dexamethasone implant (0.7 mg) was injected intravitreally, followed by bevacizumab (1.25 mg) in the superior temporal quadrant. Immediately after the injections, the patient’s visual acuity was tested to prevent visual loss due to increased IOP spikes. For 5 days after the procedure, patients used topical antibiotics (e.g., moxifloxacin). Twenty-four hours after the procedure, all patients were contacted via phone and questioned about symptoms of eye pain and worsening of visual acuity. They were then revaluated after 30 days and after 60 days.

**Statistical Analysis**

Descriptive analysis was used to present the demographic and clinical data. Data with normal distribution are presented as the mean ± the standard deviation (SD). Data with non-normal distribution are presented as the median (interquartile range). The independent samples t test was used to compare continuous normally distributed variables (i.e., IOP and visual acuity), whereas the Wilcoxon (signed-rank) test was used to compare non-normally distributed variables (i.e., macular thickness). Each parameter was compared at three time points: baseline (i.e., before treatment), and 30 days and 60 days after intravitreal injection of dexamethasone intravitreal implant and bevacizumab. MedCalc software (MedCalc Inc., Mariakerke, Belgium) was used for computerized statistical analysis. Statistical significance was set at P < 0.05.

## RESULTS

Twenty patients (representing 20 eyes) with a mean age of 73.4 ± 8.9 years were included in the study. Table 1 presents the patients’ baseline characteristics. Before study entry, 11 eyes were pseudophakic, five eyes were phakic, and four eyes had a cataract. At the time of injection (i.e., baseline), the BCVA was 0.758 ± 0.42 logMAR and improved significantly to 0.51 ± 0.33 logMAR at 1 month and to 0.5 ± 0.34 logMAR at 2 months (P ≤ 0.03).

**Figure 1:**
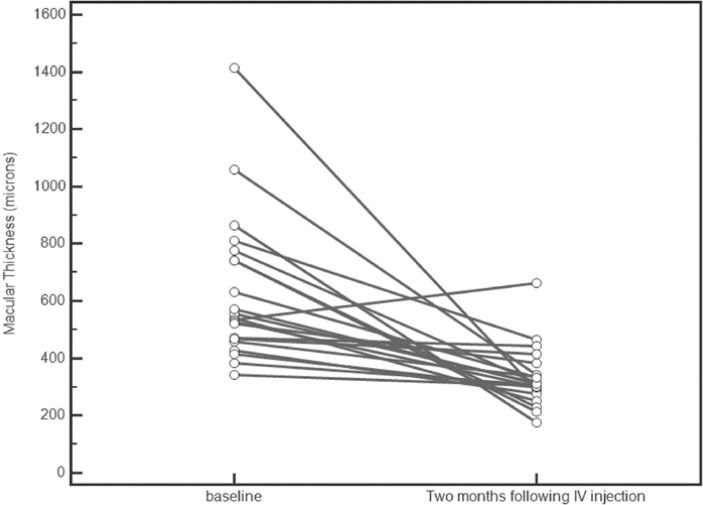
The graph shows the change in macular thickness after simultaneous treatment during 2 months of follow up. IV, intravitreal.

The median baseline central macular thickness (CMT) was 542 µm (interquartile range, 466–751 µm). The median CMT decreased significantly to 321 µm (interquartile range, 288–381 µm) at 1 month and to 310 µm (interquartile range, 286 – 354 µm) at 2 months (P ≤ 0.0002). Figure 1 presents the change in macular thickness after simultaneous treatment for 2 months. There was one case of ME recurrence after 2 months; the patient was treated with an anti-VEGF injection.

After 2 months, the mean IOP increased from 14.9 ± 2.29 mmHg (at baseline) to 16.5 ± 2.99 mmHg (P = 0.04). Two (10%) eyes experienced increased IOP. However, in both patients, the IOP was controlled with two topical anti-glaucoma medications. Deterioration of a pre-existing cataract occurred in 2 (10%) eyes, and 1 patient required cataract surgery. There were no treatment-related cases of iris and angle neovascularization, retinal detachment, vitreous hemorrhage, intraocular lens damage, ocular hypotony, endophthalmitis, or glaucoma surgery.

## DISCUSSION

Many investigators have compared the use of an anti-VEGF drug, followed by a low-release implant containing a corticosteroid drug to treat ME with intervals ranging from 2 weeks to 3 months between procedures (1-3). Few studies have used the concomitant therapy. Our data demonstrated the beneficial effects and the lack of serious adverse effects with this treatment modality. In this study, a simultaneous intravitreal injection of bevacizumab and a dexamethasone implant safely reduced ME and improved visual acuity during a 2-month follow up.

**Table 1:** Demographic and baseline characteristics, anatomic and functional results, and complications in patients with macular edema treated with a combined therapy

**Patient No.**	**Sex**	**Age**	**Diagnosis**	**Baseline**	**Month 1**	**Month 2**	**Baseline**	**Month 1**	**Month 2**	**Complications**
**1**	F	68	DME	810	517	464	0.7	0.5	0.4	None
**2**	M	73	DME	460	381	335	1	1	1	Cataract progression
**3**	M	82	CRVO	1416	296	290	1.3	0.6	0.6	None
**4**	F	66	DME	427	280	277	0.5	1.3	1.4	Cataract progression
**5**	M	50	DME	542	244	250	0.6	0.3	0.3	None
**6**	F	85	BRVO	556	291	313	0.7	0.7	0.7	None
**7**	M	66	CRVO	741	230	231	1.3	0.2	0.1	None
**8**	F	73	DME	472	438	433	0.4	0.3	0.3	None
**9**	F	68	CRVO	344	307	304	1.1	1.1	0.8	None
**10**	M	70	DME	534	451	664	0.4	0.4	0.7	Increased IOP
**11**	M	84	DME	382	321	309	0.3	0.3	0.3	None
**12**	F	81	CRVO	1058	342	344	HM	CF	CF	None
**13**	F	70	DME	468	410	414	0.3	0.3	0.3	None
**14**	M	78	CRVO	865	175	176	1.3	0.8	0.8	None
**15**	M	74	BRVO	776	355	314	0.6	0.3	0.3	None
**16**	F	80	BRVO	414	312	298	0.4	0.2	0.2	None
**17**	F	63	BRVO	743	217	214	1.7	0.5	0.4	None
**18**	M	71	DME	528	308	301	0.6	0.3	0.2	Increased IOP
**19**	F	87	BRVO	522	384	385	1	0.6	0.6	None
**20**	F	80	BRVO	572	321	310	0.2	0.1	0.1	None

BCVA: best-corrected visual acuity; BRVO: branch retinal vein occlusion; CF: counting fingers; CMT: central macular thickness; CRVO: central retinal vein occlusion; DME: diabetic macular edema; HM: hand movement; IOP: intraocular pressure; logMAR: logarithm of the minimum angle of resolution.

**Figure 2:**
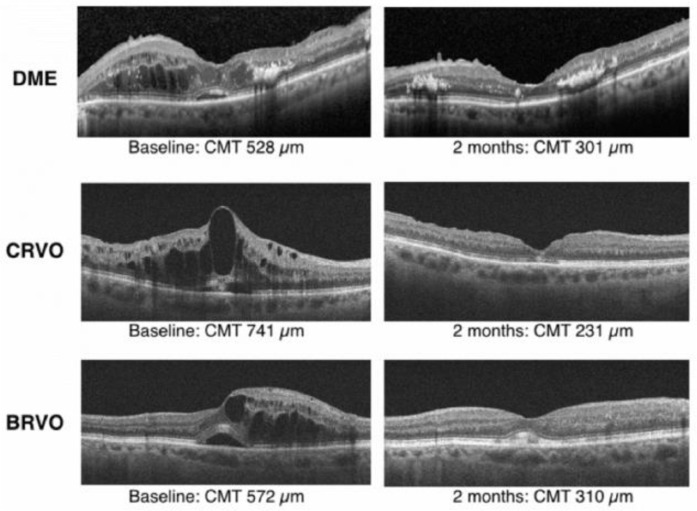
Optical coherence tomography retinal images of representative study eyes with DME, CRVO, and BRVO at baseline and after treatment with dexamethasone implant and bevacizumab. Optical coherence tomography indicates central macular edema. BRVO, branch retinal vein occlusion; CMT, central macular thickness; CRVO, central retinal vein occlusion; DME, diabetic macular edema; OCT, optical coherence tomography.

This study is among the first studies to investigate this treatment regimen. During the first month, the central macular thickness decreased significantly (P = 0.0001), and this reduction remained significant at the end of the second month (P = 0.0002; Figure 2). Visual acuity improved significantly after 30 days and 60 days of follow up (P ≤ 0.03).

Our data compares favorably with the data of previous studies. For example, Michael et al. (2) demonstrated progressive mean visual improvement within 2 weeks in individuals with vein occlusion after they received combined therapy. Raj et al. (3) showed that 1 month after combined therapy, edema in patients with diabetic ME dramatically improved, and this improvement was sustained for at least 3 months. The increase in IOP after 2 months was significant (P = 0.04); however, we believe that the mean elevation of IOP (1.6 mmHg) could be attributed to variations in measurements without clinical impact. The increase in IOP in our patients may not have increased, as described in other studies (8-13), because of the limited follow up period of our investigation. Many patients who have barriers to a monthly follow-up regimen or who have disease recurrence may become a significant burden. For these patients, simultaneous treatment may result in a fewer number of procedures and may be more beneficial and comfortable (14). In the present series, the concomitant therapy was well tolerated.

A limitation of our study is that it was retrospective, nonrandomized, and uncontrolled. The follow up lasted only 2 months; therefore, it was impossible to estimate the long-term efficacy and safety of the dexamethasone implant and the need for reinjection. In addition, because of the lack of a control group, we cannot rule out the possibility that the reduction in the macular thickness may be associated with better systemic control, especially in patients with diabetic ME. It is possible that the low rate of the progression of cataracts could be related to the short follow-up period and reduced number of injections. In summary, simultaneous therapy of dexamethasone implant and bevacizumab for macular edema may be an attractive treatment regimen with an acceptable safety profile. Further trials are indicated to better define the long-term efficacy and adverse effects of this regimen.
